# Immunoradiation Therapy for End-Stage Undifferentiated Cervical Cancer That Restored Sensitivity to Chemotherapy and Resulted in the Disappearance of the Cancer

**DOI:** 10.7759/cureus.57144

**Published:** 2024-03-28

**Authors:** Hisashi Nagai

**Affiliations:** 1 Human and Environmental Studies, Tokai University, Kanagawa, JPN; 2 Oncology, Ginza Phoenix Clinic, Tokyo, JPN

**Keywords:** liver metastatis, gyanecologic oncology, regenerative therapy, oncoimmunology, cancer immunotherapy

## Abstract

Among cervical cancers, small cell undifferentiated carcinoma is rare. Because of its rapid progression, the prognosis is extremely poor. During the course of cisplatin-based chemotherapy for stage Ⅳ small cell undifferentiated carcinoma of the cervix, the patient developed drug resistance, and standard treatment was no longer feasible. Therefore, immunoradiotherapy was administered to activate anticancer immunity. Surprisingly, the cancer drug sensitivity was restored, and cisplatin was again successful, and the cancer disappeared. In addition, the activation of cancer-specific immunity maintained the disappearance of the cancer. It should be noted that immunoradiotherapy not only increases anti-cancer immunity but may also contribute to overcoming cancer drug resistance.

## Introduction

Among cervical cancers, small cell undifferentiated carcinoma is rare. Because of its extremely rapid progression, median survival is estimated to be less than one year [1,2]. Undifferentiated cancers are prone to acquire drug resistance to chemotherapy. Overcoming drug resistance is a very important issue in cancer treatment because the inability to administer chemotherapy means the end of standard treatment. In this regard, radiotherapy has been shown to have the potential to restore the sensitivity of cancer to drugs, which is a promising approach for a different purpose than the elimination of cancer. However, there have been few clinical reports [3,4].

Recently, immunoradiation therapy, which combines radiation therapy and immunotherapy, has attracted much attention. In immunoradiation therapy, irradiation increases the immunogenicity of cancer and enhances the anticancer immune response, thereby increasing the clinical efficacy of immunotherapy [5].

In the case of this paper, immunoradiotherapy was administered to a patient with end-stage undifferentiated cervical cancer. As a result, the drug sensitivity was restored, and the ineffective anticancer drug became effective again. The cancer disappeared. The patient was able to continue the disappearance of cancer due to the increase in anti-cancer immunity.

## Case presentation

A woman in her 50s underwent a wide hysterectomy, bilateral adnexectomy, and pelvic lymph node dissection after discovering cervical cancer two years earlier. Pathological examination revealed small cell undifferentiated carcinoma.

Tumor markers such as SCC, CEA, and CA125 were all negative. This patient's cervical cancer was of a nature not accompanied by elevated tumor markers. Cisplatin (63 mg, 1w x 4 times) was administered as postoperative chemotherapy and 50Gy/25f as postoperative radiation therapy. Three weeks after completion of treatment, right supraclavicular fossa lymph node metastasis, paratracheal lymph node metastasis, and liver metastasis were detected by CT. Therefore, paclitaxel 280 mg/w and cisplatin 94 mg/w were started, and radiotherapy was simultaneously added at 50Gy/25f to the right supraclavicular fossa and paratracheal lymph node metastases. However, one month later, evaluation CT showed acute progression of liver metastases. Thus, chemotherapy was discontinued.

**Figure 1:**
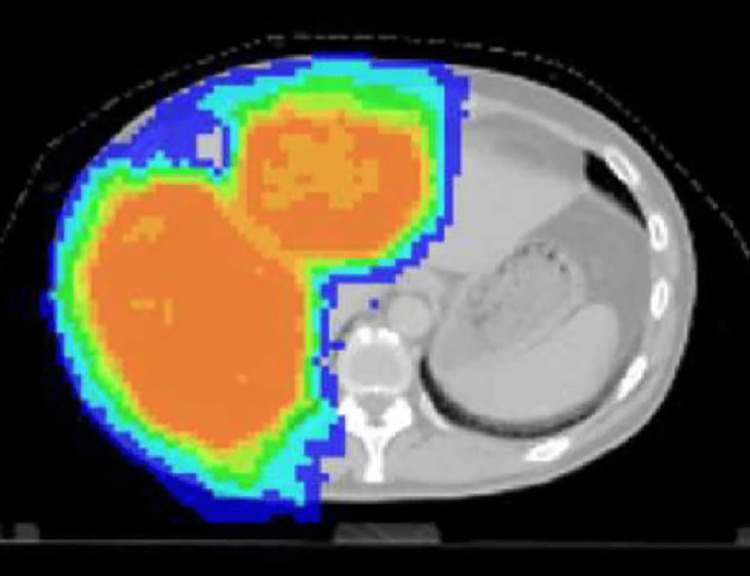
CT of the irradiated area before the start of WT1-DC The orange area is the main irradiated area. Cervical cancer metastases occupied 60% of the liver.

**Figure 2:**
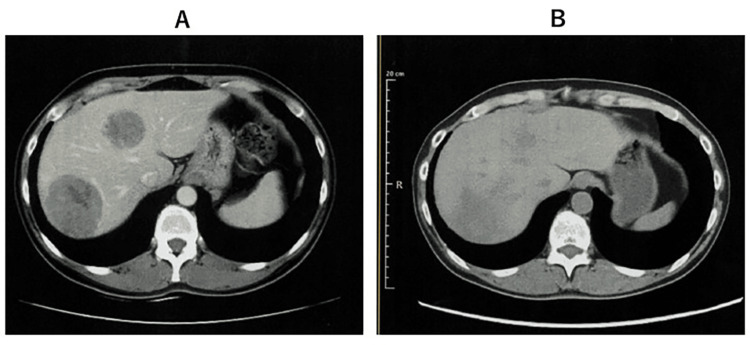
Change of liver metastases over time (A) Abdominal CT after one month of irradiation showing marked shrinkage of liver metastases. (B) Abdominal CT after the fifth dose of WT1-DC showing the disappearance of liver metastases.

At this time, the liver had two large metastases, both of which together occupied 60% of the liver. The patient received radiotherapy (25Gy/5f) for the entire liver metastases at another hospital (Figure 1). One month later, a CT scan showed that the liver metastases had shrunk markedly (Figure 2A). Therefore, on the recommendation of the radiologist, Wilms tumor 1 pulsed dendritic cell vaccine therapy (WT1-DC) and nivolumab 20 mg were started as immunoradiotherapy once every two weeks. 4.06 × 10^7^ dendritic cells in total per dose were injected subcutaneously into the right and left parainguinal lymph nodes.

**Figure 3:**
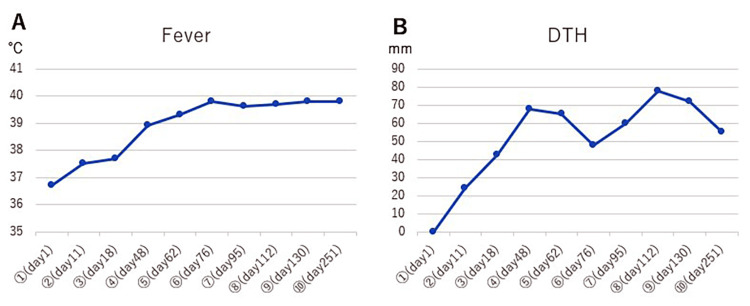
WT1-DC-induced immune response (A) Change in maximum body temperature within three days after WT1-DC administration. The temperature exceeds 39℃ after the fourth dose. (B) Change in DTH size within three days after WT1-DC administration. Diameter mean value remains high after the fourth dose. DTH: delayed type hypersensitivity.

After the second administration of WT1-DC, the patient began to have fever on the night of the day of administration. After the fourth administration, fever of 39°C or higher was observed (Figure 3A). However, the fever returned to normal within three days without drugs.

To evaluate the induction level of WT1-specific cytotoxic T cells (WT1-CTLs), 0.1 ml of WT1 antigen was inoculated on the palmar side of the forearm, and the size of the resulting erythema (delayed type hypersensitivity, DTH) was measured. After the fifth dose of dendritic cell vaccine (67 days after the first WT1-DC injection), CT showed that the liver metastases had disappeared (Figure 2B).

**Figure 4:**
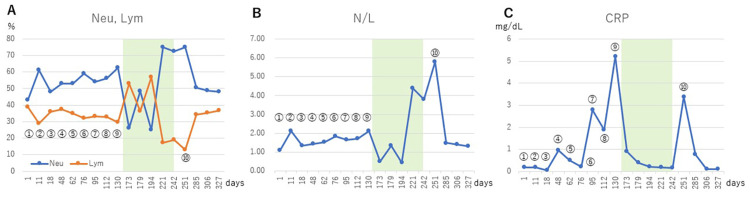
Changes in immune profile status Changes over time in indicators of immune response after initiation of WT1-DC. Numbers ⓵ to ⑩ indicate the number of WT1-DC inoculations. Green area indicates duration of CDDP and PEM administration. Nue: Nuetrophil, Lym: Lymphocyte, N/L: Nuetrophil/Lymphocyte ratio, CRP: C-reactive protein.

On the other hand, up to the seventh injection of WT1-DC, the ratio of neutrophils to white blood cells was in the 50% range and lymphocytes in the 30% range, and the ratio of neutrophil/lymphocyte ratio (N/L) was in the 1 range, indicating a good immune profile status (IPS) (Figures 4A, 4B). In blood tests, CRP levels began to rise from the fourth injection of WT1-DC (Figure 4C). However, after the seventh WT-DC, the percentage of neutrophils gradually increased, the percentage of lymphocytes decreased, and the N/L increased.

**Figure 5:**
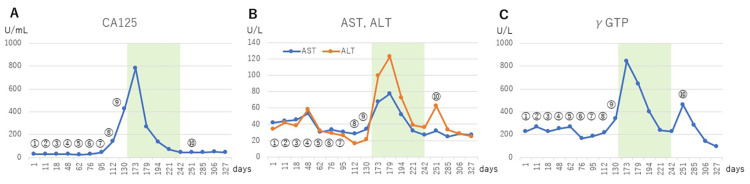
Changes in tumor markers and liver enzymes Changes in CA125 (A) and liver enzymes (B, C) after the start of WT1-DC. Numbers ⓵ to ⑩ indicate the number of WT1-DC inoculations. CA125: carbohydrate antigen 125, AST: aspartate aminotransferase, ALT: alanine aminotransferase, γGTP: γ-glutamyl transpeptidase.

At the same time, the blood CA125 level began to rise, and from the ninth WT-DC, AST, ALT, and γGTP also began to increase (Figures 5A-5C). At this time, five metastases in the lungs, pleural effusions on both sides, and recurrent liver metastases were observed (no imaging data). The patient developed dyspnea and was admitted to the hospital.

Although there were no chemotherapeutic agents to administer according to cancer practice guidelines, chemotherapy physician challenged to start cisplatin 94 mg/w and pembrolizumab 200 mg/3w. As a result, tumor markers returned to normal level after two months, and the lung and liver metastases had disappeared.

After the 84-day of cisplatin and pembrolizumab treatment period, a sharp rise in CRP was observed, so a 10th dose of WT1-DC was administered on day 251 (Figure 4C). Blood samples taken at the time of the 10th dose showed worsening of liver function, elevation of CRP, and worsening of IPS, but these values subsequently normalized. At day 357, there was no recurrence of cancer, and her physical condition was still good with performance status 2.

## Discussion

In this case, the patient received radiotherapy and WT1-DC after the cancer became resistant to cisplatin. When cisplatin was used again for subsequent cancer progression, the cancer disappeared, and tumor markers normalized. This phenomenon indicates that cancer may lose its resistance to chemotherapy once acquired, either by radiotherapy WT1-DC, or both. This patient's cervical cancer was negative for all tumor markers, but at the time of progression after radiotherapy and WT1-DC treatment, it was accompanied by a sharp increase in CA125. This indicates that the nature of the cancer was altered by the therapeutic intervention.

Because irradiation damages cancer genes, the resistance genes acquired by the cancer are also damaged. This may alter the intensity of the cancer's drug resistance [6].

On the other hand, irradiation increases the expression of HLA class 1 molecules on the membrane of cancer cells. The increased presentation of cancer antigens enhances anti-cancer immunity. It has also been reported that cancer cell death activates anti-cancer immunity by exposing cancer antigens hidden inside the cells [3,4]. The first step for cancer-specific immunity is the recognition by dendritic cells of cancer antigens presented on the surface of cancer cells. It has also been reported that HLA class 1 expression is critical for enhancing anti-cancer immunity [7]. The combination of irradiation and dendritic cell therapy is an excellent way to reactivate the weakened anti-cancer immune system in advanced cancer. Radiation therapy and immunotherapy are a good combination and are being studied under the name “immunoradiation therapy” [5].

WT1 is one of the common cancer antigens. According to a 2009 report by the National Cancer Institute in the U.S., WT1 ranked first among 75 common cancer antigens for an overall score of 9 items, including therapeutic efficacy, immunogenicity, cancer specificity, and expression level [8]. Cervical cancer also expresses WT1 in more than 80% of cases [9]. In this case, WT1 was used as the target antigen for the dendritic cell vaccine. WT1-CTL induction was considered sufficient because WT1-DC administration resulted in high fever and a well-defined DTH of 50-80 mm in mean diameter.

Radiation should increase HLA class 1 presenting WT1 antigens on the surface of cancer cells. WT1-CTLs presented from WT1-DCs recognize WT1 on the surface of the cancer cell membrane, which makes it easier to attack cancer cells. In this case, nivolumab was used in combination with WT1-DC, and pembrolizumab was used during subsequent cisplatin therapy. The combination of immune checkpoint inhibitors may have increased the efficacy of WT1-DC, and it can be considered that high anti-cancer immune function was maintained through the continuation of immunotherapy over a long period of time.

On the other hand, blood tests on day 9 after cisplatin and pembrolizumab administration was completed showed a sharp rise in CRP, AST, ALT, and γGTP. In addition, a neutrophil% increase, lymphocyte% decrease, and N/L increase were observed. These indicate a worsening of IPS. However, after the 10th administration of WT1-DC, these values quickly normalized and have remained. It is likely that the chemotherapy damaged immune cells, which reduced the anti-cancer immune function, resulting in a temporary exacerbation of the cancer, which was restored by the additional vaccination.

In this case, CRP was also elevated from the fourth injection of WT1-DC. CRP is an independent indicator of cancer activity [10]. It should be noted that elevated CRP levels persisted prior to elevated CA125 and worsening IPS. Elevated CRP in the absence of elevated tumor markers or other abnormal hematologic findings may indicate a worsening of the cancer.

## Conclusions

The effect of immunotherapy such as immune checkpoints is known to improve overall survival, which is called a “tail plateau” because of the shape of the survival curve. In contrast to chemotherapy, which is characterized by short-term tumor shrinkage and improved survival, immunotherapy is characterized by its long-term anticancer effects. During the course of chemotherapy, cancers often develop drug resistance. However, the addition of immunoradiation therapy may change the nature of the cancer and make it sensitive to the drug again. Also, sustained activation of anti-cancer immunity can inhibit cancer growth in the long term. In advanced cancers that are difficult to eliminate completely, immunoradiation therapy can increase the number of chemotherapy options as well as activate the immune system. These effects may be extremely effective in extending the life expectancy of patients with advanced cancer.
